# 

**Published:** 1895-08-24

**Authors:** 


**CADBURY'S COCOA**

"Of absolute purity and freedom from alkali."

—*Braithwaite.*

**CADBURY'S COCOA**

"The Typical Cocoa of English Manufacture Absolutely Pure."—*The Analyst.*

LIBRARY Index S. G. O.

### THE HOSPITAL

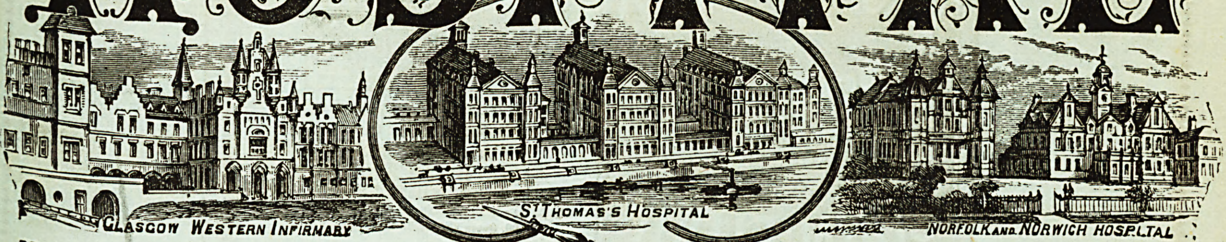

No. 465. Vol. XVIII.

SATURDAY, AUGUST 24TH, 1895.

**WITH EXTRA NURSING SUPPLEMENT.**
[Registered at the General Post Office as a Newspaper for transmission in the United Kingdom and Abroad.]

**PRICE TWOPENCE.**

Per Annum, 10s. 6d., post free.

Monthly Parts, 6d.; post free, 8d.

Ditto per Annum, 8s. 6d., post free.

